# Low dose aspirin in the prevention of recurrent spontaneous preterm labour – the APRIL study: a multicenter randomized placebo controlled trial

**DOI:** 10.1186/s12884-017-1338-0

**Published:** 2017-07-14

**Authors:** Laura Visser, Marjon A. de Boer, Christianne J. M. de Groot, Tobias A. J. Nijman, Marieke A. C. Hemels, Kitty W. M. Bloemenkamp, Judith E. Bosmans, Marjolein Kok, Judith O. van Laar, Marieke Sueters, Hubertina Scheepers, Joris van Drongelen, Maureen T. M. Franssen, J. Marko Sikkema, Hans J. J. Duvekot, Mireille N. Bekker, Joris A. M. van der Post, Christiana Naaktgeboren, Ben W. J. Mol, Martijn A. Oudijk

**Affiliations:** 1 Department of Obstetrics and Gynecology, VU University Medical Center, De Boelelaan 1118, 1081 HZ Amsterdam, The Netherlands; 2 Department of Obstetrics and Gynecology, Birth Centre Wilhelmina Children Hospital, University Medical Center Utrecht, Heidelberglaan 100, 3584 CX Utrecht, The Netherlands; 3 Department of Obstetrics and Gynecology, Academic Medical Center, Meibergdreef 9, 1105 AZ Amsterdam, The Netherlands; 4 Department of Obstetrics and Gynecology, Leiden University Medical Center, Albinusdreef 2, 2333 ZA Leiden, The Netherlands; 5 Department of Health Sciences and the EMGO Institute for Health and Care Research, Faculty of Earth and Life Sciences, Vrije Universiteit Amsterdam, De Boelelaan 1085, 1081 HV Amsterdam, The Netherlands; 6 Department of Obstetrics and Gynecology, Robinson Research Institute, University of Adelaide, 72 King William St, North Adelaide, SA 5006 Australia; 7 Department of Obstetrics and Gynecology, Maxima Medical Center in Veldhoven, De Run 4600, 5504 DB Veldhoven, The Netherlands; 8 Department of Obstetrics and Gynecology, Maastricht University Medical Center, P. Debyelaan 25, 6229 HX Maastricht, The Netherlands; 9 Department of Obstetrics and Gynecology, Radboud University Medical Center, Geert Grooteplein-Zuid 10, 6525 GA Nijmegen, The Netherlands; 10 Department of Obstetrics and Gynecology, University Medical Center Groningen, Hanzeplein 1, 9713 GZ Groningen, The Netherlands; 11 Department of Obstetrics and Gynecology, Hospital Group Twente, Zilvermeeuw 1, 7609 PP Almelo, The Netherlands; 12 Department of Obstetrics and Gynecology, Erasmus Medical Center, ‘s- Gravendijkwal 230, 3015 CE Rotterdam, The Netherlands; 13 Department of Neonatology, Isala Clinic, Dokter van Heesweg 2, 8025 AB Zwolle, The Netherlands; 14 Julius Center for Health Sciences and Primary Care, University Medical Center Utrecht, Heidelberglaan 100, 3584 CX Utrecht, The Netherlands

**Keywords:** Pregnancy, ‘Spontaneous recurrent preterm birth’, SPTB, Preterm birth, Preterm labour, PTB, Prevention, Reduction, Aspirin, Acetylsalicylic acid, ASA

## Abstract

**Background:**

Preterm birth (birth before 37 weeks of gestation) is a major problem in obstetrics and affects an estimated 15 million pregnancies worldwide annually. A history of previous preterm birth is the strongest risk factor for preterm birth, and recurrent spontaneous preterm birth affects more than 2.5 million pregnancies each year. A recent meta-analysis showed possible benefits of the use of low dose aspirin in the prevention of recurrent spontaneous preterm birth. We will assess the (cost-)effectiveness of low dose aspirin in comparison with placebo in the prevention of recurrent spontaneous preterm birth in a randomized clinical trial.

**Methods/design:**

Women with a singleton pregnancy and a history of spontaneous preterm birth in a singleton pregnancy (22–37 weeks of gestation) will be asked to participate in a multicenter, randomized, double blinded, placebo controlled trial. Women will be randomized to low dose aspirin (80 mg once daily) or placebo, initiated from 8 to 16 weeks up to maximal 36 weeks of gestation. The primary outcome measure will be preterm birth, defined as birth at a gestational age (GA) < 37 weeks. Secondary outcomes will be a composite of adverse neonatal outcome and maternal outcomes, including subgroups of prematurity, as well as intrauterine growth restriction (IUGR) and costs from a healthcare perspective. Preterm birth will be analyzed as a group, as well as separately for spontaneous or indicated onset. Analysis will be performed by intention to treat. In total, 406 pregnant women have to be randomized to show a reduction of 35% in preterm birth from 36 to 23%. If aspirin is effective in preventing preterm birth, we expect that there will be cost savings, because of the low costs of aspirin. To evaluate this, a cost-effectiveness analysis will be performed comparing preventive treatment with aspirin with placebo.

**Discussion:**

This trial will provide evidence as to whether or not low dose aspirin is (cost-) effective in reducing recurrence of spontaneous preterm birth.

**Trial registration:**

Clinical trial registration number of the Dutch Trial Register: NTR 5675. EudraCT-registration number: 2015-003220-31.

## Background

Preterm birth (<37 weeks of gestation) is the most common cause of neonatal morbidity and mortality worldwide. Worldwide in 2010 an estimated 14.9 million neonates were born preterm of whom 1.6 million were born < 32 weeks of gestation [1].

Preterm birth accounts for 70% of all neonatal mortality and for 40% of childhood neurological morbidity [2] resulting in a tremendous impact on both the child and its parents. The effects of preterm birth are not restricted to the neonatal period. Prematurity is a risk factor for developmental problems later in life. The risk of complications is directly proportional to the severity of prematurity; adverse neonatal outcome declines from 77% at 24–27 weeks to less than 2% beyond 34 weeks [2, 3].

Gestational age at birth determines to a large extent the health prospects of the neonate. The increased physical and mental health problems for preterm born children result in increased health costs, leading to a considerable economic burden for society [4].

One third of preterm births are due to medical intervention because of maternal or fetal complications, while two third of preterm births occurs after spontaneous onset of the delivery [5]. The strongest risk factor associated with preterm birth is a previous preterm birth, with a relative risk (RR) of 6.0 (95% CI 4.1 – 8.8) if the preterm birth was < 32 weeks in the index pregnancy and RR 4.8 (95% CI 3.9–6.0) if birth was between 32 and 36 weeks [6]. Currently, the mostly used strategy for prevention of recurrent spontaneous preterm birth is the administration of progestagens, either natural progesterone or 17 Alpha-hydroxyprogesterone. A Cochrane review on the subject concluded that the use of progesterone is associated with benefits in infant health and showed a reduction of preterm birth less than 37 weeks (10 studies; 1750 women; pooled RR 0.55, 95% CI 0.42 to 0.74) [7]. In the largest trial on the subject a reduction from 55% to 36% (RR 0.66; 95% CI 0.54–0.81) was observed [8]. A recent publication of a large trial showed that vaginal progesterone was not associated with reduced risk of preterm birth or composite neonatal adverse outcomes [9]. Thus, the literature on the subject is ambiguous. Despite the use of progestagens, at least one third of the women will have a recurrent spontaneous preterm birth, suggesting that multiple underlying mechanisms contribute to its pathogenesis [10].

An increasing body of evidence suggests that utero-placental ischemia plays a major role in the genesis of spontaneous preterm labour. Placental vascular pathology and placental bed pathology are common findings in women with a spontaneous preterm birth [10–13]. Also, abnormal angiogenic/anti-angiogenic profile in maternal plasma is seen in a subset of patients with spontaneous preterm birth [14], and increased resistance at midtrimester Doppler measurement of uterine artery flow provides an increased risk of spontaneous preterm birth [15]. In addition, women with a spontaneous preterm birth have an increased risk of developing cardiovascular disease later in life [16, 17]. These findings suggest an overlap with other ischemic placental diseases such as preeclampsia.

In the light of the possible similar underlying mechanism, preventive measures for ischemic placental disease might also be effective in preventing recurrent spontaneous preterm birth. The most successful intervention in the prevention of recurrent preeclampsia is low dose acetylsalicylic acid (aspirin or ASA). Aspirin acts as a thrombocyte aggregation inhibitor and it reduces prostaglandin synthesis through inhibition of the enzyme cyclooxygenase, thereby having an anti-inflammatory effect. A recent meta-analysis showed a reduction of preeclampsia with a RR 0.47 (0.34-0.65) if aspirin was initiated before 16 weeks of gestation [18]. Interestingly, a marked reduction in total preterm births was also observed with a RR of 0.22 (0.0–0.49). This reduction in preterm birth could not be fully explained by the reduction of indicated preterm birth by clinicians for medical reasons, suggesting also a decrease in spontaneous preterm births associated with the use of aspirin. A recently published Individual Participant Data (IPD) meta-analysis specifically looked at rates of spontaneous preterm birth in women receiving antiplatelet agents for the indication of high risk of preeclampsia [19]. Women assigned to aspirin treatment compared to placebo or no treatment had a lower risk of spontaneous preterm birth <34 weeks (RR 0.86, 95% CI 0.76–0.99) and <37 weeks (RR 0.93, 95% CI 0.86–0.996).

Furthermore, in other groups of patients where aspirin is used to improve pregnancy outcomes there is also evidence suggesting an effect on spontaneous preterm birth.

A recent IPD meta-analysis on preconception started low-dose aspirin treatment in IVF patients found a lower, though not statistically significant, incidence of preterm birth in women with a singleton pregnancy after adjustment for hypertensive pregnancy complications (OR 0.52, CI 0.16–1.7) [20].

A Dutch randomized clinical trial on aspirin and low molecular weight heparins in patients with recurrent miscarriage showed the lowest incidence of preterm birth in the aspirin group of 1.6%, versus 10.1% in the combined (aspirin and Low Molecular Weight Heparin (LMWH)) group and 4.3% in the placebo group [21]. Another recent randomized clinical trial, randomizing women with one or two recent pregnancy losses between low dose aspirin and placebo, started preconception and continued until 36 weeks of gestation, showed a trend towards a lower spontaneous preterm birth rate with a RR of 0.45, 95% CI 0.19 to 1.08 [22, 23].

Aspirin is a safe intervention in pregnancy as a recent review commissioned by the 'U.S. Preventive Services Task Force' concluded [24]. In view of the potential common underlying etiology of ischemic placental disease such as preeclampsia and spontaneous preterm birth, the proven preventive effect of aspirin on preeclampsia and the promising results in prevention of spontaneous preterm birth in RCTs on IVF, miscarriage and preeclampsia, our hypothesis is that aspirin administered before 16 weeks of gestation onwards will reduce recurrent spontaneous preterm birth. As this treatment is at present not routine practice and as this hypothesis has never been tested in a randomized clinical trial in patients with a previous spontaneous preterm birth, we designed a randomized clinical trial on the subject. The effect of aspirin on spontaneous preterm birth is tested best in a high-risk population, being women with a previous spontaneous preterm birth.

## Methods/design

### Aims

The objective of the study is to assess the (cost-)effectiveness of low dose aspirin in comparison with placebo in preventing preterm birth when initiated in early pregnancy in women with a previous spontaneous preterm birth.

### Design and setting

A multicenter, randomized, double blinded, placebo controlled trial will be performed within the Dutch Consortium for Healthcare Evaluation and Research in Obstetrics and Gynecology (NVOG Consortium 2.0, www.studies-obsgyn.nl).

### Participant/eligibility criteria

Patients 18 years of age or older are eligible in case of a singleton pregnancy between 8 and 16 weeks of gestation with a previous spontaneous preterm birth in a singleton pregnancy, defined as birth at a gestational age between 22 and 37 weeks. Spontaneous preterm birth is defined as a preterm birth following spontaneous contractions with intact membranes or preterm birth after spontaneously ruptured membranes. Exclusion criteria include women with previous indicated preterm births for maternal reasons such as preeclampsia or HELLP-syndrome or for fetal reasons such as IUGR, other indications for aspirin use, thrombocytopenia/thrombocytopathy, major fetal malformations in current pregnancy or in previous spontaneous preterm birth.

### Procedures, recruitment, randomization and collection of baseline data

Eligible women will be identified by the local research coordinators and/or the staff of participating hospitals. Women eligible for the trial will be counseled by ‘good clinical practice’ (GCP) trained doctors, midwifes or research nurses. After counseling and reading the patient information form, patients will be asked for written consent. We will provide patient information in Dutch as well as in English. After informed consent, patient data will be entered in a web-based database, which will also facilitate randomization.

Women will randomly be double-blindly allocated to either aspirin administration or placebo administration in a 1:1 ratio: both participant and healthcare worker will be unaware of what group the women is allocated. Randomization will be performed centrally with the use of a random 2 : 4 block design.

At study entry, baseline demographic, past obstetric and medical history will be recorded into the web-based Case Report Form (CRF) that is accessible through a closed part of a central website, OpenClinica. Details of delivery, maternal and neonatal assessments during pregnancy or post-partum are recorded in the CRF. The collected data will be coded and processed with adequate precautions to ensure patient confidentially.

### Intervention

Eligible women will be randomized by a web-based central randomization system to low dose aspirin 80 mg one tablet a day or placebo one tablet a day, preferably taken in the evening. Administration will be initiated between 8 and 16 weeks of gestation and will be continued up to 36 weeks of gestation or up to delivery, whatever comes first. Other preventive measures such as administration of progesterone, cerclage or pessary will be provided according local protocols for prevention of preterm birth derived from the national guidelines [25].

### Follow up

All details on delivery, maternal and neonatal assessments and admission during pregnancy are recorded in the CRF that is accessible through the website. Mortality and morbidity will be specified for the mother and the child until date of discharge from hospital or 3 months of corrected age. We plan long term follow-up of the children at age of 24 and 60 months to examine (neuro-) developmental outcomes.

### Outcome measures

The primary outcome measure will be preterm birth, defined as birth at a GA of less than 37 weeks. The most important secondary outcome is a composite of poor neonatal outcome (including bronchopulmonary dysplasia (BPD), periventricular leucomalacia (PVL) > grade 1, intraventricular hemorrhage (IVH) > grade 2, necrotizing enterocolitis (NEC) > stage 1, retinopathy of prematurity (ROP), culture proven sepsis and perinatal death). The individual components of the composite outcome will also be assessed separately.

The diagnosis of BPD will be made at 36 weeks of postmenstrual age, if a neonate has received supplemental oxygen for at least 28 days. Classification will take place using an oxygen reduction test [26]. PVL > grade 1 and IVH > grade 2 will be diagnosed by repeated cranial ultrasound by the neonatologist, according to the Papille classification [27, 28]. NEC stage >1; will be diagnosed and classified by the Bell staging criteria. ROP will be classified conform the International Classification of Retinopathy of Prematurity [29]. Culture proven sepsis is defined as the combination of clinical signs and a positive blood culture. Distinction will be made between: early onset sepsis (EONS) and late onset sepsis (LONS). For EONS clinical suspicion and positive blood culture suffices for a diagnosis. LONS will be diagnosed according to the definition of Stoll [30] that also requires a CRP blood level of >10 mg/L. Finally, perinatal death is defined as death of a fetus or neonate at any time between a gestational age of ≥ 16 weeks and discharge.

Additional secondary neonatal outcomes are number of days on ventilation support (mechanical ventilation and/or respiratory support by Continuous Positive Airway Pressure (CPAP)), infant respiratory distress syndrome (IRDS) that requires treatment with surfactant, patent ductus arteriosus (PDA) that requires medical or surgical treatment, cerebellar bleeding, days of admission on the NICU, convulsions, asphyxia, proven meningitis, pneumothorax and total days in hospital until 3 months corrected age.

Furthermore, preterm birth rates at ≤ 28 weeks, ≤ 32 weeks, ≤ 34 weeks of gestation will be assessed as a group and will also be analyzed separately as spontaneous, indicated or as a combination. Growth restriction will be analyzed and birth weight will be assessed both continuous and < p10. Separate assessment of birth weight will take place on fetal growth chart [31] and is defined as estimated fetal weight < P10.

Maternal outcomes include maternal side effects, maternal mortality, hospital admissions in general and specifically due to threatened preterm labour with need for treatment with tocolysis and or corticosteroids for antenatal fetal lung ripening. Maternal morbidity due to hypertensive disease in pregnancy will be defined according to the ACOG-guideline ‘hypertension in pregnancy’ [32]. Pregnancy-induced hypertension will be defined as: development of new hypertension ≥ 140/90 mmHg measured twice after 20 weeks of gestation. Preeclampsia will be defined as: hypertension ≥ 140/90 mmHg measured twice after 20 weeks of gestation and proteinuria (protein ≥ 300 mg/24 h) or thrombocytopenia (thrombocytes < 150 x 10e9/L), impaired liver function or renal insufficiency. Eclampsia will be defined as: seizures in a pregnancy complicated by preeclampsia. HELLP syndrome will be defined as: as the presence of hemolysis, elevated liver enzymes and low platelets with or without the presence of proteinuria of hypertension. Placental abruption will be defined as: clinical diagnosis of (partial) detachment of the placenta from the uterine wall [32].

Other maternal outcomes will be defined as follows: gestational diabetes as onset of diabetes after 20 weeks of gestation. The diagnosis of pulmonary edema will be based on clinical findings, thromboembolic disease will include both deep vein thrombosis and pulmonary embolism, maternal infection or inflammation will be a clinical diagnosis and major ante- or post-partum hemorrhage (blood loss ≥ 1 liter) will also be assessed.

The core-outcomes for research concerning interventions to prevent preterm birth as defined by the CROWN-initiative [33] will be assessed where possible to ensure that data from trials that assess prevention of preterm birth can be compared and combined, for example in future meta-analyses.

Costs will be measured from a healthcare perspective using case record forms until 3 months of corrected age of the infant. Costs will include costs of outpatient visits, hospital admissions, and the delivery. For the valuation of health care utilization, standard prices published in the Dutch costing guidelines will be used. Alternatively, hospital administration data or tariffs will be used if necessary. Medication use will be valued using prices of the Royal Dutch Society for Pharmacy.

Hypothesis generating subgroup analyses will be performed for women with a previous preterm birth before and after 30 and 34 weeks GA, a history of preterm birth starting with contractions with intact membranes versus preterm premature rupture of membranes (PPROM), women additionally treated with progestagens versus no additional treatment, women with a short cervix (<25 mm before 24 weeks) in the current pregnancy and women who started using the study-medication at less than 12 weeks versus 12-16 weeks of gestation.

### Statistical issues

#### Data-analysis

Data will be analyzed according to the intention-to-treat principle. Participants will be kept in the study regardless of whether they decide to discontinue the study medication unless they explicitly withdraw permission to use their data. Because the results are all to be extracted from the medical records of the participants, it is not expected that there will be missing data. Best effort will be made to retrieve the data. If still there is missing data, this will be mentioned along with the reason. Baseline characteristics of the groups will be presented using percentages, means with 95% confidence intervals (CI) and medians with interquartile ranges, where appropriate. No interim analysis for efficacy will be performed, however an independent Data Safety and Monitoring Committee will be monitoring patient safety every 6 months, starting 9 months after the inclusion of the first patient.

The main outcome variable, ‘reduction in recurrent preterm birth’, will be assessed by comparing the event rates in the two groups using a chi-square test with a *p*-value of 0.05. The main outcome will be presented as absolute and relative risks (along with 95% confidence intervals) and numbers needed to treat (if applicable).

The secondary outcome is poor neonatal outcome. Categorical secondary outcomes will be assessed in the same way as the primary outcome, except that Fisher’s exact test will be used where appropriate. For continuous secondary outcomes, differences between groups will be assessed with the student’s *t*-test if the outcome is normally distributed and with a non-parametric Mann-Whitney *U* test if skewed. These outcomes will be presented per group as means with standard deviation, geometric means with 95% CI, or as median with interquartile range, whichever appropriate.

A Kaplan–Meier plot of gestational age at delivery will be presented and the difference gestational age at birth between the study arms will be tested with the log-rank test. Along with this plot, the percentage non-delivered at 28, 30, 32, and 34 weeks will be presented per arm.

#### Sample size

A reduction of recurrent preterm birth from 36 to 23% (a reduction of 35%) can be detected if 406 patients are recruited (203 in each arm, power of 80%, (alpha-error 0.05). Because estimates are not consistent, the assumption of the occurrence of recurrent preterm birth was made based on the largest trial performed on prevention of preterm birth using progesterone [8].

#### Economic analysis

The economic evaluation will be performed from a healthcare perspective. The primary outcome will be costs (savings) per pre-term birth prevented. Missing cost and effect data will be imputed using multiple imputation. Cost and effect differences will be estimated using bivariate regression models with adjustment for confounders and effect modifiers if necessary. Bias-corrected accelerated bootstrapping (5000 replications) will be used to assess statistical uncertainty. Incremental cost-effectiveness ratios (ICERs) will be calculated by dividing the difference in costs by the differences in primary and secondary outcomes between the groups. Cost-effectiveness acceptability curves showing the probability that the intervention is cost-effective in comparison with usual care for a range of different ceiling ratios will also be estimated [34]. In a budget impact analysis results from the economic evaluation will be extrapolated to the national level. The economic analysis will be published in a separate paper.

#### Serious adverse events

A data safety monitoring board is established to perform ongoing safety surveillance. Serious adverse events will be reported to the board every 6 months along with the treatment arm. No interim analysis for efficacy will be performed.

#### Confidentiality and data security

Linking personal data with randomization number can only be done in the local clinics. Each participating clinic receives a login name and password to gain access to the web-secured database. The access is restricted to the database of the clinic to which the password and login name belongs. Full access to the entire database is restricted to some members of the research staff.

## Discussion

Recurrent spontaneous preterm birth remains a large contributor to the perinatal morbidity and mortality as a result of preterm birth, with a need for additional preventive methods. Based on research that points in the direction of placental ischemia as a (partial) cause of spontaneous preterm birth, interventions that improve the placentation may be effective in reducing the number of (recurrent) spontaneous preterm birth. This study is designed to provide evidence on the (cost-) effectiveness of low dose aspirin in comparison with placebo in preventing preterm birth in women with a history of spontaneous preterm birth. Low dose aspirin is considered safe in pregnancy and is already widely embedded in the obstetric practice for other indications. Therefore, if aspirin is proven to be effective in reducing recurrent spontaneous preterm birth, it would be easy to implement in daily practice.

## Abbreviations

(S)PTB: (Spontaneous) preterm birth
ASA: Aspirin
BPD: Bronchopulmonary dysplasia
CRF: Case report form
GA: Gestational age
GCP: Good clinical practice
HELLP: Hemolysis elevated liver enzymes and low platelets
IUGR: Intrauterine growth restriction
IVF: In vitro fertilization
IVH: Intraventricular hemorrhage
LMWH: Low molecular weight heparin
NEC: Necrotic enterocolitis
NICU: Neonatal intensive care unit
PE: Preeclampsia
PVL: Periventricular leukomalacia
ROP: Retinopathy of prematurity
